# The long-term consequence of salivary contamination at various stages of adhesive application and clinically feasible remedies to decontaminate

**DOI:** 10.1007/s00784-020-03307-3

**Published:** 2020-06-09

**Authors:** Pooja Nair, Nicoleta Ilie

**Affiliations:** Department of Operative Dentistry and Periodontology, Ludwig-Maximilians University, Goethestraße 70, 80336 Munich, Germany

**Keywords:** Saliva contamination, Saliva decontamination, Universal adhesive, 2-step self-etch adhesive, Bulk-fill resin composite, Shear bond strength

## Abstract

**Objective:**

To analyse the bond quality in dentine post-ageing after salivary contamination and decontamination at different stages of dental adhesive application.

**Materials and methods:**

A total of 1120 human dentine specimens were randomly allocated to 14 groups for four intervals (*n* = 20) to be treated with a self-etching (SE) and universal (U) adhesive. The saliva contamination and decontamination were implemented after surface preparation, after primer application (for SE) and after adhesive curing. The decontamination groups were either rinsed and air-dried or rinsed, air-dried and reapplied with adhesive. They were stored (37 °C, distilled water) for four intervals (1 week, 1 month, 3 months and 1 year) and subjected to shear bond strength (SBS) test at a crosshead speed of 0.5 mm/min.

**Result:**

One-way ANOVA with Tukey’s test (*α* = 0.05) revealed significant reduction in SBS in all the groups in U adhesive compared with the control group at 1 week (*p* < 0.0001) and in SE when the contamination took place after primer application. However, decontamination improved the SBS in SE but not in U adhesive. The univariate analysis confirmed significant influences (*p* < 0.0001) seen by treatment procedure (${\eta}_{\mathrm{p}}^2$=0.075), type of adhesive (${\eta}_{\mathrm{p}}^2$ = 0.328), ageing (${\eta}_{\mathrm{p}}^2$ = 0.13), experimental groups (${\eta}_{\mathrm{p}}^2$ = 0.518), and the stage of influence (${\eta}_{\mathrm{p}}^2$ = 0.60).

**Conclusion:**

Saliva contamination is detrimental after primer application in SE but, decontamination regained the SBS and maintained it over time. In U adhesive, SBS deteriorated over time irrespective of the contamination.

**Clinical relevance:**

Salivary contamination showed different influences on SBS at various stages of restoration with contemporary dental adhesives.

## Introduction

Adhesive systems have momentously transformed dentistry, allowing dental procedures that were considered impossible in the past without fashioning retentive features in cavity preparations and losing healthy tooth structure [1, 2]. Adhesively bonding the restorative composite is a multistep process and involves technique-sensitive materials. This leads to numerous instances for various factors to influence and manipulate the procedure and impair the quality of the final restorations. In an ideal situation, it is fundamental to have an operatory field isolated from unfavourable factors; however, this is not always attainable in daily dental practice. There are various situations where the isolation protocol can be susceptible to a breach, especially when the operative site is near or at the gingival margin, the patient is unwilling, teeth are malpositioned or have cervical lesions [3]. The contaminants can interfere with the infiltration of the adhesive monomers into the dentinal tubules that are required to offer sufficient bonding and may result in reducing the quality of the bond in the long term. Saliva is one such element existing in the oral cavity, which has a high probability of contaminating the surface to be restored. It has been observed that an acid conditioned tooth surface absorbs salivary constituents and decreases the surface energy and end up being detrimental for bonding [4]. In a clinical study of self-etching adhesives, the SEM evaluation of restored teeth revealed that saliva contamination resulted in shorter resin tags which were easily pulled off from the dentinal tubules while applying a load, resulting in interfacial fracture [5]. In another study, the SEM evaluation confirmed that saliva contamination did not inhibit hybrid layer formation, but, it reduced the adaptation of the restorative material to bonded surfaces [6].

In a few of the prior studies, it was observed that 2-step etch-and-rinse adhesive was relatively less vulnerable to salivary contamination [7–10]. Nevertheless, the etching process completely eliminates the smear layer and is not considered the golden standard for dentine bonding. The self-etching adhesive, on the other hand, does not eliminate the entire moisture but modifies the smear layer to form a hybrid layer [11], making it ideal for bonding to the dentine. The literature about the effect of saliva contamination on the bond strength facilitated by self-etch adhesives is ambiguous. Some studies conveyed that the self-etching adhesives are more vulnerable to salivary contamination in the dentine [5, 12–17]; in contrast, few studies ascertained that there was no significant difference due to salivary contamination in bond quality while bonding to the dentine [18–20].

The universal adhesive can be used either in etch-and-rinse or self-etching mode, and the previous studies on the salivary contamination have conveyed that saliva contaminations can be deleterious. One study [21] observed that contamination significantly reduced the bond strength and another study [22] stated that the reduction was more pronounced when the contamination occurred before light curing than after. In both the studies, decontamination involving reapplication of the adhesive restored the bond strength.

It is also observed that the salivary contamination does not have the same influence at various stages of the bonding process [23]. The earlier findings had also suggested that when the contaminated surface was subjected to some sort of decontamination procedure like rinsing the saliva or reapplying the adhesive system, the restoration attained improved adhesion [7, 13, 17, 22, 24–29]. However, there is no consistency in the procedure of decontamination, and findings thus varied [7] also; the long-term implication after the decontamination procedure is undetermined.

Although immediate studies on the influence of bond strength post-contamination have been discussed in great detail, it is also essential to understand the consequences of contamination of these modern formulations together with clinically possible decontamination methods post-ageing. A simple-to-use, all-in-one adhesive system is appealing; however, no long-term data exist on the performance of universal adhesive systems on the consequence after salivary contamination and decontamination.

Comprehending the altering microstructure of the interface and its defects over time remains a challenge at hand. Therefore, the purpose of this study was to evaluate the long-term effects of salivary contamination on the bond strength of the self-etching and universal adhesive and also to find the clinically possible remedies using decontamination procedures at various stages of application. The null hypotheses that were evaluated in this study are that the SBS in dentine is not affected by (a) the type of adhesive, (b) ageing (1 week, 1 month, 3 months and 1 year), (c) salivary contamination, (d) decontamination methods and (e) the stage of salivary contamination.

## Materials and methods

### Specimen preparation

**Fig. 1:**
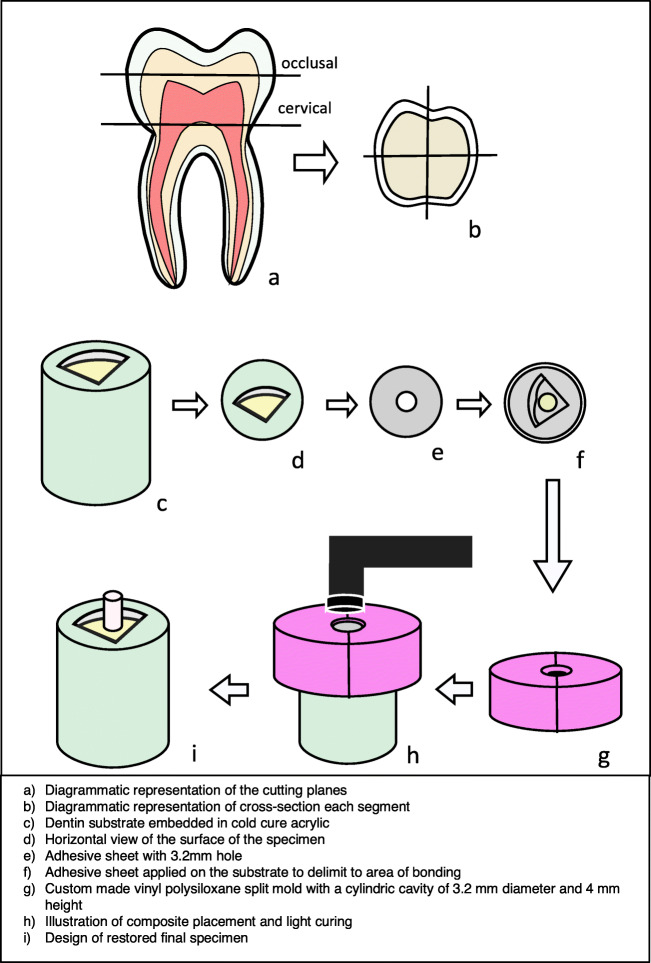
Diagrammatic representation of the overview of specimen preparation

**Fig. 2:**
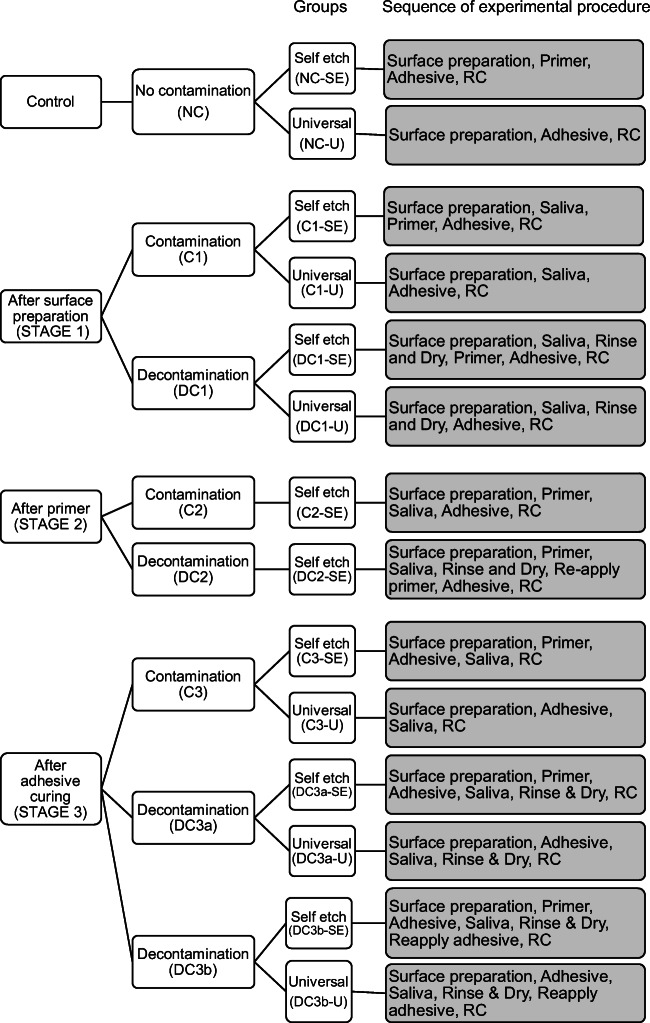
Description of the experimental groups

Extracted carious-free human third molars were collected and stored in dilute sodium azide solution at 4 °C. They were thoroughly cleaned and were sectioned mid-coronally, parallel to the occlusal plane using a low speed saw (Isomet, Buehler, Lake Bluff, IL, USA) to obtain two dentine segments labelled as “occlusal” and “cervical” (Fig. 1a). The obtained segments were further divided into 2 or 4 parts depending on the size of the tooth, ensuring that there is enough (> 3.2-mm diameter) dentine to bond (Fig. 1b). A total of 1120 dentine substrates obtained were embedded in cold-curing methacrylate resin (Technovit 4004, Heraeus Kulzer, Germany) with the help of stainless-steel cylindrical moulds (Fig. 1c). The substrates were wet ground with 600-grit silicon carbide grinding paper (Leco, St. Joseph, USA) and a grinding system (Exakt 400 cs, Norderstedt, Germany) to obtain and flat dentinal surface (Fig. 1d). They were then randomly allocated into 56 groups (*n* = 20), 14 subgroups for four intervals, 1 week (1 W), 1 month (1 M), 3 months (3 M) and 1 year (1 Y). Care was taken to avoid the specimens obtained from the same tooth be included in one group. They were treated with two adhesives: Clearfil SE Bond 2(SE) and Clearfil Universal (U) (Table 1). A thin adhesive strip with a circular hole (3.2-mm diameter) (Fig. 1e) was placed on the prepared surface, limiting the region to be bonded (Fig. 1f). The exposed dentine surface was then treated with the adhesive according to the group allocated (Fig. 2).

**Table 1:** Material composition and description

Material (acronym) manufacturer (lot no.)	Type of material	Composition	Instructions for use
Clearfil SE Bond 2 (SE) Kuraray Noritake (000031)	2-step self-etching adhesive	Primer •2-Hydroxyethyl methacrylate •10-Methacryloyloxydecyl dihydrogen phosphate •Hydrophilic aliphatic dimethacrylate •dl-Camphorquinone •Accelerators •Water •Dyes Adhesive •Bisphenol A diglycidylmethacrylate •2-Hydroxyethyl methacrylate •10-Methacryloyloxydecyl dihydrogen phosphate •Hydrophobic aliphatic dimethacrylate •Colloidal silica •dl-Camphorquinone •Initiators •Accelerators	Apply primer and leave for 20 s. Dry with mild air. Apply bond. Make a uniform bond film using a gentle airflow. Light cure for 10 s
Clearfil Universal (U) Kuraray Noritake (000017)	Universal adhesive	Adhesive •Bisphenol A diglycidylmethacrylate •2-Hydroxyethyl methacrylate •ethanol •10-Methacryloyloxydecyl dihydrogen phosphate •Hydrophilic aliphatic dimethacrylate •Colloidal silica •dl-Camphorquinone •Silane coupling agent •Accelerators •Initiators •Water	Apply bond liquid and rub for 10 s. Blow mild air to make a uniform bond film. Light cure for 10 s
Admira Fusion X-tra (AFX) Voco (1537600)	Bulk fill resin composite	Matrix: ormocer Fillers: silicon dioxide	Dispense an increment of 4-mm and light cure for 20 s

### Experimental procedure

Groups with no contamination (NC) served as control and were treated as per the manufacturer’s instructions (Table 1). The contamination (C) and decontamination (DC) treatment occurred in three stages: stage 1, after surface preparation; stage 2, after primer (only for SE); and stage 3, after adhesive curing (Fig. 2). The sequence of experimental procedure for each group is expressed in Fig. 2.

Fresh unstimulated human saliva from a single individual was collected. It was made sure to be collected at least 1 hour after the consumption of any food or drink and just before the substrate preparation. The contamination and decontamination procedures simulated the clinical situation during the process of restoration. In all the contamination and decontamination groups (C & DC), the salivary contamination was done with one drop (0.025 ml) of saliva for 20 s. In stage 1 contamination groups (C1), the surfaces were contaminated after surface preparation with saliva (20 s) and air-dried (5 s), and in the decontamination group (DC1), saliva was applied (20 s), and then rinsed with water (10 s) and air-dried (5 s). In stage 2 (only in SE), the saliva was applied (20 s) after the primer application (C2) and was decontaminated by rinsing with water (10 s), air-dried (5 s) and the primer was reapplied (20 s) (DC2). In stage 3, the saliva was applied after the adhesive system was cured (C3) and was decontaminated in two ways, either by only rinsing with water (10 s) and air-drying (5 s) (DC3a) or by rinsing with water (10 s), air-drying (5 s) and reapplying the bonding liquid and curing (10 s) (DC3b).

Except for the experimental modifications wherever mentioned, the rest of the procedures in both SE and U adhesives were as per the manufacturer’s instructions (Table 1) using the self-etch bonding method and cured for 10 s (Bluephase; Ivoclar-Vivadent; Schaan, Lichtenstein) with a radiant emittance of 1316 ± 5.1 mW/cm^2^ as measured with MARC simulator (BlueLight Analytics Inc., Halifax, Canada). A custom-built vinyl polysiloxane split mould (Regisil PB, Dentsply Caulk; USA) with a cylindrical cavity (3.2 mm in diameter and 4 mm in height) (Fig. 1g) was positioned on the specimen. An ormocer-based bulk-fill resin composite, Admira fusion x-tra (Voco, Cuxhaven, Germany) was then placed in one 4-mm increment, followed by polymerizing it for 20 s (Bluephase; Ivoclar-Vivadent; Schaan, Lichtenstein) (Fig. 1h).

The prepared specimens (Fig. 1i) were stored vertically immersed in distilled water at 37 °C for four different time intervals (1 week, 1 month, 3 months and 1 year). The distilled water was periodically changed every week without disturbing the specimens. After storing the specimens for the predetermined durations, they were subjected to SBS test with a broad chisel head in a universal testing machine (MCE 2000ST; Quicktest Prüfpartner GmbH, Langenfeld, Germany) at a constant crosshead speed of 0.5 mm/min until fracture. Subsequently, the loaded force at fracture was recorded. Post-fracture, the diameter of the fractured specimens was measured to a precision of 0.01 mm using a digital micrometre scale at two perpendicular positions, and then, the bonded area was determined using the mean diameter. The SBS was calculated by dividing the force required to fracture the specimen by the bonded area.

### Fracture pattern analysis

The fractured fragments were then carefully examined with a 10× magnification. Failure modes were classified as an adhesive failure (failure along the adhesive interface), mixed failure (failure along the adhesive together with the failure of the resin composite or dentine) or cohesive failure (> 80% of the failure involving the resin composite, dentine or both).

### Statistical analysis

The SBS results were statistically analysed (Version 25.0; IBM SPSS Statistics. USA) for normality and homogeneity of variance using the Kolmogorov-Smirnov test and Levene’s test, respectively. The SBS data of individual experimental groups over time were evaluated using a one-way analysis of variance (ANOVA) with the Tukey’s HSD post-hoc test (*α* = 0.05). The univariate analysis (general linear model with partial eta squared (${\eta}_{\mathrm{p}}^2$)) (*α* = 0.05) was used to analyse the influence of the parameters; treatment procedures, type of adhesive, ageing, stage of influence and experimental groups on the bond strength. Additionally, SBS of specimens obtained from occlusal and cervical parts of the tooth were compared within each control (NC) experimental group in order to assess a possible influence of dentine substrate obtained from different areas of the tooth.

To assess the reliability of each experimental group, Weibull analysis using linear regression was performed based on the SBS data to determine the Weibull modulus and characteristic strength (*n* = 20), at a confidence level of 95%. The expression of Weibull distribution ${P}_{\mathrm{f}}\left(\upsigma \right)=1-\exp \left[-{\left(\frac{\upsigma}{\upsigma_0}\right)}^{\mathrm{m}}\right]$where *P*_f_ is the probability of fracture at applied stress, σ is the measured strength, *σ*_0_ is the characteristic strength at which probability of fracture is 63.21% and *m* is the Weibull modulus [30]. The double logarithm of this expression gives $\mathrm{lnln}\ \left(\frac{1}{1-\mathrm{P}}\right)=\mathrm{mln}\upsigma -\mathrm{mln}{\upsigma}_0$. By mapping$\mathrm{lnln}\ \left(\frac{1}{1-\mathrm{P}}\right)$ versus ln(σ), a linear upward gradient m and its intersection with the x-axis gives the logarithm of the characteristic strength (σ_0_). The scatter in the computed Weibull parameters as well as the bias were analysed and compared with results at a 95% confidence level using ${P}_{\mathrm{f}}=\frac{\left(\mathrm{i}-0.5\right)}{n}$ estimator [31].

## Results

**Fig. 3:**
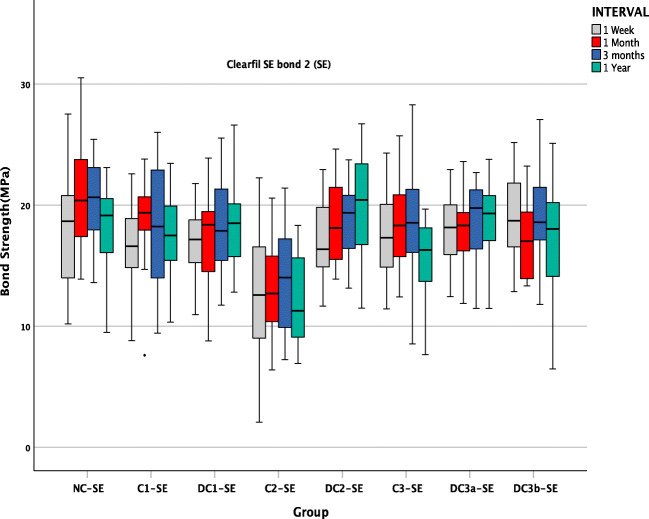
SBS of all groups in SE adhesive over time

**Fig. 4:**
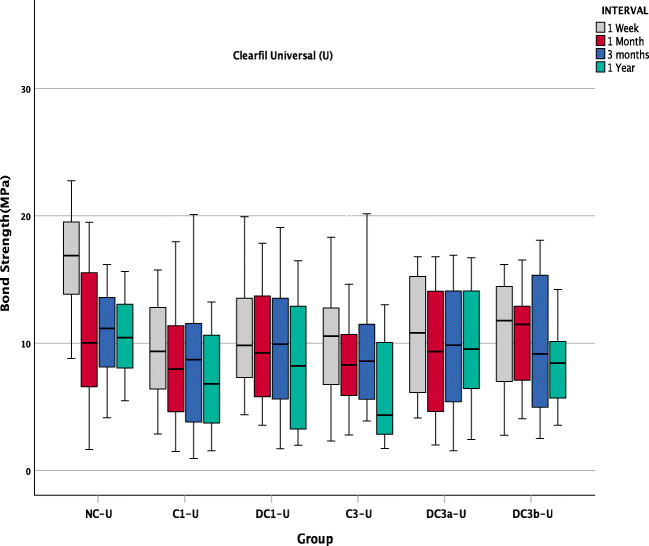
SBS of all groups in U adhesive over time

SBS data of SE adhesive (Fig. 3) and U adhesive (Fig. 4) depict the ageing behaviour of different groups pre- and post-contamination with saliva. It can be observed that among the control groups, there is a drastic reduction in SBS in the NC-U, while a stable bond strength was observed in NC-SE groups all throughout 1 year of the ageing period. Although immediate bond strength comparison of the control groups of both adhesives NC-SE and NC-U showed no significant difference (*p* = 0.186) at 1 week, there was a significant difference in the SBS of the control group over time.

All the control groups in U adhesive had a significant reduction in SBS compared with the control group of 1 week storage (NC-U) (*p* < 0.0001) (Fig. 4) whereas there was no significant influence of ageing on the SBS in dentine on the control groups of SE adhesive (NC-SE) (*p* = 0.517) (Fig. 3).

At the 1-week storage period, it can be observed that the salivary contamination significantly reduced the SBS (C1-U, C3-U) in U adhesives compared with the NC-U, and the decontamination procedures (DC1-U, DC3a-U and DC3b-U) could not restore the SBS to control levels (NC-U)(Fig. 4). Nonetheless, in the 1-month and 3-month intervals, the influence of contamination, although lower, was not significantly different as compared with the SBS values of the control (NC-U) group. The lowest mean SBS was recorded for the group C3-U (6.27 ± 4.06 MPa) at 1-year interval which was significantly lower SBS compared with NC-U (10.51 ± 3.11 MPa) at 1 year (Fig. 4).

SE group showed a statistically significant reduction in the SBS only when the contamination occurred after the application of primer (C2-SE) in all the intervals of ageing (1 week,1 month,3 months and 1 year) (Fig. 3), although decontaminating the surface by rinsing, drying and re-applying the primer and adhesive considerably improved the SBS and was similar to the control group levels at all the intervals of ageing (DC2-SE) (Fig. 3).

The general linear model with partial eta squared statistics revealed that there was significant influence seen by the stage of influence (${\eta}_{\mathrm{p}}^2$ = 0.600, *p* < 0.0001), experimental groups (${\eta}_{\mathrm{p}}^2$ = 0.518, *p* < 0.0001), type of adhesive (${\eta}_{\mathrm{p}}^2$ = 0.328, *p* < 0.0001), ageing (${\eta}_{\mathrm{p}}^2$ = 0.130, *p* = 0.003) and the treatment procedure (${\eta}_{\mathrm{p}}^2$ = 0.075, *p* < 0.0001). The part of the tooth (occlusal or cervical) exhibited no significant influence (*p* = 0.527) on the SBS when the control groups (NC) for both the adhesives across the ageing process was observed.

**Fig. 5:**
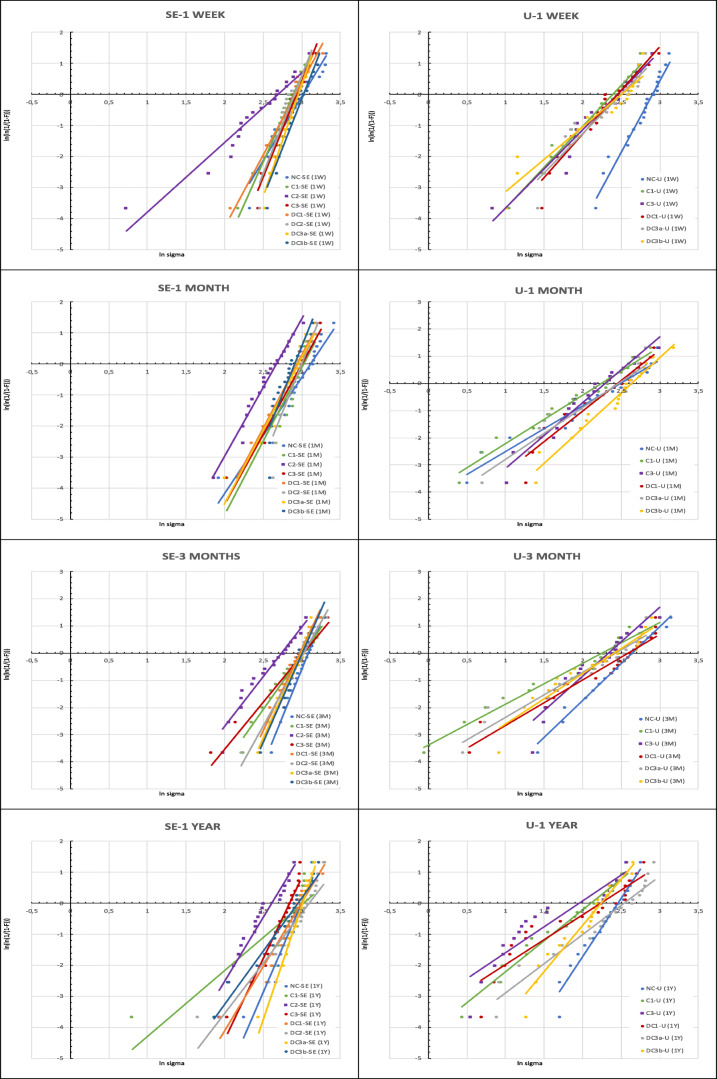
Weibull plot of all groups of SE and U adhesive over time

The Weibull analysis data of SE adhesive and U adhesive (Fig. 5) illustrate the Weibull modulus (m) at 95% confidence level and characteristic strength (σ_0_) of each experimental group over time. The *m* values of U adhesive were lower than SE in all the intervals irrespective of stages of contamination. The *m* values in SE adhesive varied from 2.12 ± 0.3 to 7.39 ± 0.09, and in U adhesive, they ranged from 1.50 ± 0.11 to 4.60 ± 0.10.

**Fig. 6:**
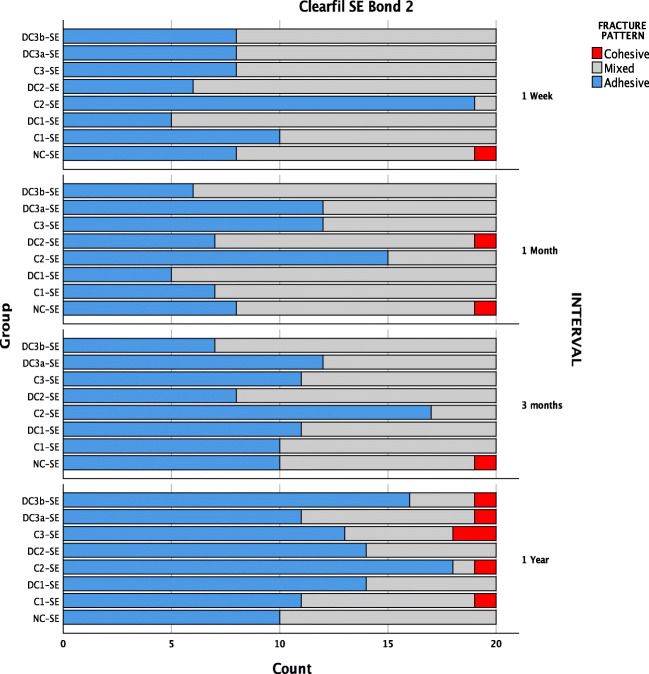
Fracture pattern observed in SE adhesive over time

**Fig. 7:**
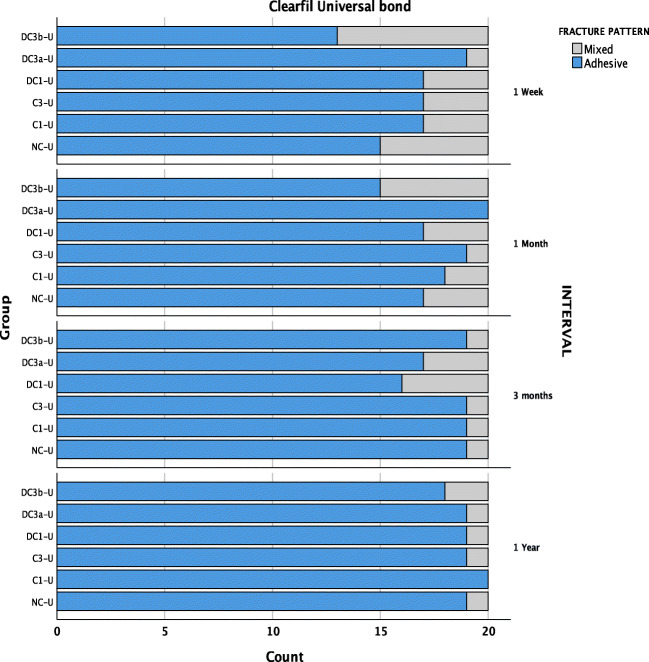
Fracture pattern observed in U adhesive over time

The fracture pattern analysis indicated a low ratio of cohesive failures (0.9%), suggesting a relatively decent set of SBS test results. The U adhesive groups showed 89% of adhesive, 11% of mixed failures and no cohesive failure (Fig. 7). Whereas, SE adhesive showed 52.6% of adhesive, 45.8% of mixed and 1.6% of cohesive failures (Fig. 6). Higher SBS values were associated with a higher ratio of mixed and cohesive failures. Mean SBS of adhesive failures (12.35 ± 5.70 MPa) was significantly lower compared with cohesive (18.72 ± 4.50 MPa) and mixed failures (18.04 ± 4.30 MPa). There were no pre-test failures observed.

## Discussion

The quintessential goal of obtaining an excellent adhesion in restorative dentistry is to produce an interface that is stable over time, guarantee adequate bond strength, good marginal seal, assure clinical durability and have minimal imperfections [32]. It is acknowledged that moisture trapped within the adhesive during polymerization may cause an inferior polymerization of the adhesive monomers [33]. Through this study, we intended to recognize the unfavourable consequences of salivary contamination on the SBS of two contemporary adhesives throughout a year in the dentine, and if the effect of contamination was found to be substantial, which stage in the adhesive application was most vulnerable to it? Furthermore, do clinically feasible decontamination procedures regain their original bond quality?

When bonded to the dentine, the U adhesive applied in self-etching mode differed significantly in their SBS compared with the SE adhesive over time. Thus, the null hypothesis that there is no difference in SBS of adhesives used for bonding to the dentine has to be rejected. The parameter “type of adhesive” showed significant influence on the SBS (${\eta}_{\mathrm{p}}^2$ = 0.328, *p* < 0.0001). Hence, the type of adhesive used is proved to be crucial when observed over time. The complexity of the dental substrate and the different characteristics of enamel and dentine necessitate the availability of diverse dental adhesive systems to contain various components that prepare the surface and interact with the different components of the tooth surface efficiently [34], and therefore, they react differently in the oral environment.

Both of the tested adhesives incorporate hydrophobic monomer, bisphenol A diglycidyl ether dimethacrylate(bis-GMA) that functions exceptionally well while bonding to enamel, whereas, the dentine is moist and inherently hydrophilic and is therefore unable to penetrate into the tubules. So, more hydrophilic monomers like 2-hydroxylethyl methacrylate (HEMA) are incorporated in the adhesive/primer to enhance wetting. However, this addition makes the adhesive layer to be more vulnerable to hydrolysis in the oral environment [35] owing to water sorption, which then behaves as a permeable membrane [36]. In addition to encouraging a reduction in bond quality between the composite and the substrate, such perviousness of the adhesive layer appears to exaggerate the hydrolysis of resin polymers and the consequential degeneration of tooth-resin bond over time [37].

The composition of universal adhesives is complex as it contains both hydrophobic as well as hydrophilic monomers in the same mixture, due to which the presence of any residual moisture can cause phase separation and result in blister formation [38]. The phase separation in BisGMA/HEMA adhesives can end up in lower bond quality as the adhesive tries to diffuse into the moist dentine matrix; the constituents separate into hydrophobic BisGMA-rich and hydrophilic HEMA-rich phases [38]. The low cross-linking potential of HEMA makes it unstable in aqueous environments which tends to degrade with exposure to oral fluids. Consequently, this phase becomes the weak point for adhesive bonding and adversely affects their durability [37].

Both the adhesives contain monomer 10-MDP, which bonds chemically to hydroxyapatite by forming stable calcium-phosphate salts without causing severe decalcification [39]. It is already known that HEMA significantly affects the chemical interaction of 10-MDP with hydroxyapatite. Although 10-MDP remained adsorbed to the hydroxyapatite crystals, it hindered nano-layering even in the lowest concentration of HEMA [40]. As the manufacturers do not provide complete data on the exact concentration of 10-MDP or its purity, it is not possible to provide an explicit statement about the influence of this monomer in terms of contamination.

The effects of ageing were evidently perceived on the U adhesive when irrespective of the treatment group all groups were considered for each interval, the combined mean SBS significantly reduced over time (*p* < 0.0001). This result has also been established in another study where the U adhesive showed a deteriorated micro-tensile bond strength after 1 year of ageing [41]. Whereas, in SE adhesive, there was no significant reduction in SBS (*p* = 0.085) over time. So, the null hypothesis that ageing does not have a significant influence on SBS is partially accepted.

The proposed null hypothesis that there will be no effect of salivary contamination for both the adhesives were rejected as there were significant differences in the SBS values exhibited by both the tested adhesives post-contamination. In the SE adhesive, the contamination was critical post-primer application (C2-SE), but the contamination at stage 1 and stage 3 (C1-SE and C3-SE) did not show any detrimental effect on the bond quality. Decreased SBS values at stage 2 significantly increased after decontamination (DC2-SE). It conveys that if noticeable salivary contamination is spotted at the priming stage, just simple water rinsing for 10 s, air-drying for 5 s and re-priming the area followed by the adhesive application will bring the bond quality to control levels and also maintain it long-term. This finding is in accordance with previous researches deliberating the effect of saliva contamination on the bond strength of self-etch adhesives which revealed that contamination after primer application significantly decreased the bond strength [13, 15, 42, 43]. The cause for this reduction is presumed to be due to the rinsing away of the hydrophilic monomer (HEMA) in the SE primer with the saliva and the water from the demineralized dentine, which may have resulted in the collapse of the collagen when the surface was air-dried after contamination. The monomers in the bonding agents could not have effectively penetrated into the dentine because of collapsed collagen. In one of the studies, the LV-SEM micrographs showed contaminants deposited on the dental surfaces, when saliva was applied after primer application, creating a physical barrier to monomer diffusion and resulting in a deteriorated adhesion [43].

In the U adhesive, contamination (C1-U and C3-U) reduced the mean SBS significantly as compared with the bond strength of control (NC-U) at 1 week or immediate testing. It was also interesting to note that the contamination after curing (C3-U) showed a drastic reduction in SBS after 1 year when compared with the control group (NC-U)The decontamination procedures (DC1-U, DC3a-U and DC3b-U) did not bring back the SBS values to the control levels. The normal pH of saliva is 6 to 7 [44]. In stage 1 contamination, the saliva present on the prepared surface could have acted as a buffer and reduced the etching capacity of monomers in the U adhesive whose pH is acidic (pH = 2) and resulted in reduced penetration into the dentinal tubules and resulting in a decreased bond quality. This observation conforms to the earlier studies [21, 22, 24, 45, 46], which established that the salivary contamination in universal adhesives could be detrimental. The influence of salivary contamination in stage 3 may be justified as the adsorption of glycoproteins onto the polymerized adhesive surface, which results in oxygen inhibition and reduced bonding capacity [24].

Decontamination by rinsing, drying and reapplying the adhesive after curing (DC3b-SE and DC3b-U) did not seem to add any benefit in short as well as long term in both the adhesives. A former study which reapplied the adhesive after decontamination also reciprocates a similar result of having no difference in the immediate bond strength of self-etching adhesives [13].

The most substantial influence on the SBS was exercised by the parameter “stage of contamination” (${\eta}_{\mathrm{p}}^2$ = 0.600) followed by the “experimental group” (${\eta}_{\mathrm{p}}^2$ = 0.518) and then the “type of adhesive”$\Big({\eta}_{\mathrm{p}}^2$ = 0.328). The stage at which the contamination or decontamination occurred was the most critical in this study. It is evident from the C2-SE group; the contamination occurring at stage 2 (after primer application) was significantly damaging to the SBS in dentine. Ageing of the specimens had a significant but relatively low influence (${\eta}_{\mathrm{p}}^2$ = 0.130) on the SBS, and treatment procedure (contamination or decontamination) of the specimens had the least influence (${\eta}_{\mathrm{p}}^2$ = 0.075).

In a study that investigated the Clearfil SE bond, which is a predecessor of the adhesive used in this study, the bond strength did not differ significantly between the saliva-contaminated group and the control group [47]. This observation was backed with the idea that the acidity of self-etch adhesives modifies and penetrates the smear layer and also breaks through the mucopolysaccharides in the saliva and develops bond strengths comparable with those obtained on noncontaminated dentine surfaces. In a few other studies, the bond strength of Clearfil SE Bond was reduced when saliva contamination occurred [5, 13, 42]. The newer and improved version (Clearfil SE Bond 2) of this well-accepted adhesive also reverberates the previous findings. However, it is crucial to consider that the stage at which saliva contamination occurs has been found to play an essential role in bond strength. This explains the lack of consensus among the various study designs with regard to its effect on dentine bond strength.

The Weibull analysis enables evaluation of data scattering by relating the probability of failure to applied stress. Defining the SBS data only with mean and standard deviation does not convey the information about the distribution of stresses at which the individual specimens failed, as these stresses could be formed due to the distribution of the flaws, like the inconsistencies or interferences in the adhesive layer, air bubbles, size and amount of filler particles, areas of inadequate conversion and separated phases within the material.

In the Weibull analysis (Table 2), the lower values of *m* are indicative of an inconsistent underlying flaw population, assuming that the material was tested accurately and failed in a brittle manner. In contrast, a higher Weibull modulus is suggestive of narrow distribution and resonates to closely placed stress values at which the specimens failed indicative of a consistent flaw. It can be seen from the Weibull plot (Fig. 5) that the slopes (Weibull moduli) indicate the strength distribution at a given interval (1 week, 1 month, 3 months and 1 year) for both the adhesives. The slopes in SE suggest that the flaw types in post-contamination groups C2 were more inconsistent, hence a lower *m* value compared with the control group (NC). It is very evident from the data that the U adhesives were less reliable compared with SE adhesives, based on their overall Weibull modulus. The deviances within the slopes in Weibull plot are not unpredicted, and they are frequently witnessed in small size sample sets. When comparing the Weibull parameters of the control groups at 1 week, it can be observed that the U adhesive (*m* = 4.60 ± 0.10, *σ*_0_ = 13.32 MPa) showed higher *m* and characteristic strength than the SE (*m* = 4.12 ± 0.13, *σ*_0_ = 12.44 MPa). Nevertheless, the reliability of the U adhesive reduced over time. In our study, since we have introduced the flaw of salivary contamination, it is evident that this variability makes the result inconsistent among the various contamination and decontamination groups.

**Table 2:** Weibull parameters of both SE and U adhesives over time

Weibull parameters									
Group		1 Week		1 Month		3 Months		1 Year	
		SE	U	SE	U	SE	U	SE	U
NC	m ± CI	4.12 ± 0.13	4.60 ± 0.10	3.75 ± 0.15	1.69 ± 0.11	7.07 ± 0.15	2.73 ± 0.07	5.81 ± 0.13	3.78 ± 0.11
	σ0 (MPa)	12.44	13.32	11.69	4.21	21.81	7.21	17.39	9.29
C1	m ± CI	5.53 ± 0.11	2.63 ± 0.07	4.75 ± 0.24	1.78 ± 0.08	4.02 ± 0.10	1.50 ± 0.11	2.12 ± 0.3	2.00 ± 0.09
	σ0 (MPa)	15.98	6.23	14.36	3.98	12.12	3.39	6.38	4.20
DC1	m ± CI	4.66 ± 0.10	2.81 ± 0.13	4.71 ± 0.09	2.27 ± 0.14	6.02 ± 0.10	1.68 ± 0.13	4.13 ± 0.14	1.60 ± 0.19
	σ0 (MPa)	13.61	6.90	13.85	5.54	17.91	4.33	12.37	3.50
C2	m ± CI	2.24 ± 0.14	-	4.42 ± 0.09	-	3.68 ± 0.13	-	4.16 ± 0.14	-
	σ0 (MPa)	6.01	-	11.8	-	10.07	-	10.86	-
DC2	m ± CI	6.28 ± 0.15	-	6.31 ± 0.18	-	5.12 ± 0.10	-	3.24 ± 0.20	-
	σ0 (MPa)	18.26	-	18.92	-	15.48	-	10.03	-
C3	m ± CI	6.27 ± 0.10	2.53 ± 0.13	4.50 ± 0.12	2.42 ± 0.11	3.47 ± 0.16	2.53 ± 0.17	5.19 ± 0.10	1.67 ± 0.21
	σ0 (MPa)	18.36	6.18	13.50	5.55	10.47	5.90	14.75	3.27
DC3a	m ± CI	7.22 ± 0.08	2.55 ± 0.16	4.73 ± 0.17	1.92 ± 0.15	6.28 ± 0.10	1.66 ± 0.13	7.39 ± 0.09	1.88 ± 0.13
	σ0 (MPa)	21.35	6.30	13.97	4.70	18.81	4.02	22.20	4.76
DC3b	m ± CI	6.38 ± 0.11	2.10 ± 0.16	6.20 ± 0.22	2.60 ± 0.11	6.52 ± 0.11	1.86 ± 0.15	3.46 ± 0.10	3.01 ± 0.13
	σ0 (MPa)	19.26	5.24	18.07	6.81	19.62	4.45	10.19	6.71

The structural and morphological differences in the dentine challenge the understanding of attaining a durable bond between adhesive resin and dentine [48]. Bond strength values have been previously reported to have been sensitive to the depth of the dentine used as a substrate, as it is influenced by the diameter of the dentinal tubules and the water content [48]. While preparing the substrate, the tooth was cut mid-coronally to obtain two portions, an “occlusal” and a “cervical” segment. These parts differed in the depth of the dentine roughly by the thickness of the diamond saw used to cut the tooth (0.270 mm). When the bond strengths obtained in the control groups (NC-SE and NC-U) of both these parts were analysed, there was no statistically significant difference (*p* = 0.527) between the occlusal or cervical parts even after 1 year of ageing in both the adhesives. This implies that the incongruity due to the difference in the dentine substrate was negligible. The substrate was prepared in such a manner in order to maximize the potential of the available dental substrate as the study required a sizeable number of specimens. For preparing the surface of the substrates, 600 grit SiC paper was used since this has been proved to be the most common and efficient way of imitating the smear layer [7, 49, 50].

In general, the failure mode distribution correlated quite well with the bond strengths of SE and U adhesives. The predominant failure mode was an adhesive failure, irrespective of saliva contamination and ageing (SE, 52.6%; U, 88.9%) (Fig. 6 and Fig. 7). The cohesive fractures with > 80% involvement of either tooth/composite were only seen with SE adhesive (0.8%) out of which 90% of the fractures occurred in the dentine which could be suggestive of the tooth to be the weaker path for the fracture propagation. The critique of the methodology is often the higher percentage of cohesive failures, because of non-uniform stress distribution. When the break occurs cohesively in the composite resin or dentine, the value attained conveys the cohesive strength. However, in the assessment of adhesive systems on substrates, the intention is to analyse the bond of the adhesive with the substrate on the dentine and not cohesive strength of other regions such as the dentine or composite resin [51]. Nevertheless, unlike micro-tensile bond strength testing, SBS is conventional and does not require vast stress-inducing procedure during specimen preparation, which often results in pre-test failures [51]. In this study, no pre-test failures were recorded.

It is not surprising to see that the SE adhesive was more resilient to hydrolytic degradation over time as they offer a distinct, hydrophobic resin layer as the final application step, unlike the U adhesive. Although bond strength happens to be an essential assessment, the lifespan of bonding is the most important indicator of clinical success. However, our findings in this study must not be generalized and should not be applied to the whole class of universal adhesives because each material features different compositions and unique modifications to achieve their functional capability.

In a clinical situation, while using 2-step SE adhesive, the most vulnerable stage for salivary contamination is after primer application. If in doubt of salivary contamination, rinsing, drying and reapplying the primer will make the adhesive last longer. Should a situation arise where clinical isolation protocol is compromised, SE adhesive could be the choice of adhesive instead of U adhesive to have more predictable results in the long term.

## Conclusion

Within the limitations of the study, the results indicate that when the universal adhesives were used in the self-etching strategy on the dentine, the bond strength deteriorated over time. Regardless of contamination or decontamination, the universal adhesive could not regain the immediate bond strength of the control group after ageing. In self-etching adhesive, the saliva contamination was most critical when the contamination occurred after primer application. Decontaminating by rinsing, air-drying and reapplying the primer regained the bond strength to control levels and maintained it over time.
